# Public Involvement to Enhance Care Home Research; Collaboration on a Minimum Data Set for Care Homes

**DOI:** 10.1111/hex.70140

**Published:** 2025-01-13

**Authors:** Anne Killett, Kerry Micklewright, Rachael Carroll, Gizdem Akdur, Emily Allinson, Liz Crellin, Kaat de Corte, Margaret Fox, Barbara Hanratty, Lisa Irvine, Liz Jones, Marlene Kelly, Therese Lloyd, Julienne Meyer, Karen Spilsbury, Ann‐Marie Towers, Freya Tracey, John Willmott, Claire Goodman

**Affiliations:** ^1^ School of Health Sciences University of East Anglia Norwich UK; ^2^ Centre for Research in Public Health and Community Care (CRIPACC) University of Hertfordshire Hatfield UK; ^3^ Academic Unit of Injury, Recovery and Inflammation Sciences School of Medicine, University of Nottingham Nottingham UK; ^4^ NIHR Applied Research Collaboration‐East Midlands (ARC‐EM) Nottingham UK; ^5^ Public Involvement Contributor; ^6^ Improvement Analytics Unit The Health Foundation London UK; ^7^ Population Health Sciences Institute Newcastle University Newcastle upon Tyne UK; ^8^ National Care Forum London UK; ^9^ Auburn Mere Watford UK; ^10^ Care of Older People City, University of London London UK; ^11^ School of Healthcare, Faculty of Medicine and Health University of Leeds Leeds UK; ^12^ NIHR Applied Research Collaboration Yorkshire and Humber (YHARC) Bradford UK; ^13^ Centre for Health Services Studies University of Kent Canterbury UK

**Keywords:** care homes, minimum data set, older people, public involvement

## Abstract

**Introduction:**

Information on care home residents in England is captured in numerous data sets (care home records, General Practitioner records, community nursing, etc.) but little of this information is currently analysed in a way that is useful for care providers, current or future residents and families or that realises the potential of data to enhance care provision. The DACHA study aimed to develop and test a minimum data set (MDS) which would bring together data that is useful to support and improve care and facilitate research. It is that utility that underscores the importance of meaningful public involvement (PI) with the range of groups of people affected. This paper analyses the involvement of family members of care home residents and care home staff through a PI Panel.

**Objectives:**

The objective for the PI activities was to consistently bring the knowledge and perspectives of family members and care home staff to influence the ongoing design and conduct of the DACHA study.

**Methods:**

The bespoke methods of PI included a dedicated PI team and a PI Panel of public contributors. Meetings were recorded and minutes agreed, resulting actions were tracked and reflections on the PI recorded. A democratic, social relations approach was used to frame the analysis.

**Results:**

A PI panel met 17 times. All meetings included both family members and care home staff. Analysis of the records and reflections developed the following themes about the operation of the PI: deepened understanding of the data environment in care homes; Influence on the pilot MDS; aiming for best research practices with care homes; personal/professional development for PI members; expectations of the project. Learning points for future research projects are developed.

**Conclusions:**

PI shaped the design and conduct of the DACHA study, grounding it in the needs and perspectives of people using and providing social care. Data research has a huge responsibility to accurately incorporate relevant public perspectives. There is an implicit assumption that records and data are objective and ‘speak for themselves’ however there can be unintended consequences from introduction of new data requirements in practice.

**Patient or Public Contribution:**

Public contributors to this manuscript include family members of older people living in care homes and staff of care homes. The wider study also involved as the public, older people living in care homes. Public contributors helped develop the project, contributed throughout the conduct of the study and some chose to be involved in preparing this manuscript.

## 1 Introduction

The Developing research resources And minimum data set for Care Homes' Adoption and use (DACHA) study aimed to develop and pilot a minimum data set (MDS) for care homes for older people in [1]. The purpose of an MDS is to enable care coordination, governance and service planning [2]. Information on care home residents in England is currently held by different organisations (e.g., care home records, health records) and not easily shared [3].

**Figure 1:**
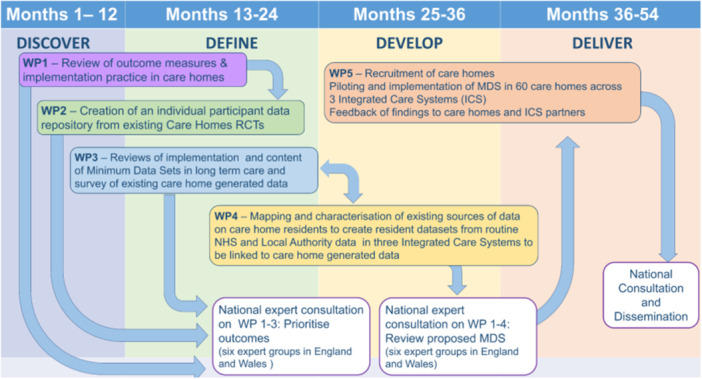
Diagram of the five work packages in the DACHA project.

The DACHA pilot MDS linked data routinely collected by health providers to data from care homes’ digital care records by identifying care home residents aged 65 or older in National Health Service (NHS) data sets [2]. The content of the pilot MDS was informed by literature reviews, stakeholder development workshops, surveys and public involvement (PI) [4, 5, 6, 7, 8, 9, 10]. The 4.5 years (2019–2024) study was divided into five related work packages (WPs) (Figure 1). Fourteen original collaborators came from nine universities, the National Care Forum, The Health Foundation (THF) and the Alzheimer's Society Research Network.

There are important stakeholders in this enterprise, and the means of involvement or engagement need to be tailored to their interest, preference, area of existing knowledge and communication needs. At the centre are the older people living in the care homes, and their families. Data recording or use has implications for those running and working in care homes. Health care organisations contribute to the care of older people in care homes [11]. Local authority adult social services departments and the Care Quality Commission (CQC) require data from care homes in their commissioning, quality assurance and regulatory roles. Care planning systems are moving from paper‐based to digital, with a government target for 80% implementation in England by 2025 [12] therefore providers of digital care planning software are also stakeholders. Effective PI could reduce the risks of negative unintended consequences from findings and recommendations [13, 14] by weighing up implications for care practices of collecting particular information which could in future be used for benchmarking.

Meaningful PI in care home research requires relationship building between researchers and PI stakeholders, and consideration of the differing perspectives and interests of stakeholders [15]. Care home research programmes have involved public representatives at each stage of the research cycle and in project management meetings [16] and in particular roles [17] but transparency about the extent, nature and influence of PI in published care home research is varied [18] with calls for more discussion of processes and evaluation [19]. Edelman and Barron [20] argued for the evaluation of PI as a component of the research process rather than as if it were a therapeutic intervention. Indeed such evaluation positions PI members as research participants, ‘othering’ them from more agentic roles in research [21]. Rather than constructing and evaluating PI as an intervention it could be conceptualised as a social practice in which researchers and the public interact and power relations are considered [22]. It is clear that research with care homes will be most effective with authentic co‐production and active collaboration between researchers and care home representatives [5].

Frith argues that PI in applied health research is best understood as an attempt to make research more democratic, with the potential to change both what is studied and the research processes, to broaden which knowledge is valued and who is involved in the production of knowledge [23]. In England, PI in health research was advocated in a national research development strategy in 1991, framed as ‘consumer’ involvement in 1996 with a publicly funded body to support it [24]. This body became INVOLVE. In 2015, the National Institute for Health and Care Research (NIHR) recommended co‐production between the public, researchers and health professionals [25]. An overview of systematic reviews of PI in clinical trials identified a range of innovative PI but oriented to consultation rather than decision‐making [26]. UK Standards for PI in Research were published in 2018 [27] providing practical examples of good PI but less consideration of the rationale for involving the public in research [28]. The remit of INVOLVE was taken into NIHR in 2020. Debates continue over PI should be part of the establishment with a remit to improve research processes or a socially transformative practice [24].

PI can be related to concepts of activism [29], active citizenship [30] and participatory research [31], with common arguments for the place of local and contextual knowledge, indigenous knowledge, experiential knowledge and situated knowledge, alongside scientific knowledge, in science policy [32]. Care homes are part of social care provision, which in the UK is argued to be in crisis and in need of a shift from a charity welfare to a rights‐based paradigm [33]. Fricker [34] proposed a concept of epistemic justice, including hermeneutic injustice, where members of groups do not have access to equal participation in the generation of social meanings and are at a disadvantage when making sense of their social experience. We argue care home staff and relatives of care home residents can be said to experience hermeneutic injustice. They are not equally included in the generation of social meaning about data and reporting requirements for care homes. Their experience is frequently marginalised. Care workers in Ontario had few opportunities to contribute their knowledge when an MDS was implemented, framed as an experience of epistemological violence [35]. We aimed to create a structure and social relations underpinned by democratic principles in which care home staff and family members' knowledge could be recognised, valued and shape the knowledge developed by the project [34]. With the findings of earlier work feeding into later stages, we aimed for iterative engagement so that stakeholders could contribute to the developing understanding. Democratic principles underpinned the DACHA approach to PI, with the aim of enabling those least heard and most affected groups to contribute their own expertise and to advocate for others. We worked to the democratic principles that public contributors would be involved in decision making [23] in order that their contributions influenced the research process.

This paper presents an analysis of the involvement of care home staff and family members of care home residents in the DACHA project, using a social relations and democratic approach and reflection on examples of key effects over the 4 years of the study. Rather than separating out and evaluating the PI component of the study we examine it in the context of building a project and outputs that are fit for purpose.

The DACHA project received ethical approvals for distinct elements of the research project:

- WP2 care home trials archive; Health, Science, Engineering and Technology ECDA—University of Hertfordshire (HSK/SF/UH/04185).
- WP3 national care home survey; Health, Science, Engineering and Technology ECDA—University of Hertfordshire (HSK/SF/UH/04301).
- WP5 care home pilot; London Queen's Square Research Ethics Committee (22/LO/0250/311711).
- National consultation 2022; Health, Science, Engineering and Technology ECDA—University of Hertfordshire (HSK/SF/UH/05009).
- National consultation 2023‐24; Health, Science, Engineering and Technology ECDA—University of Hertfordshire (HSK/SF/UH/05487).

Ethical review was not sought for this analysis of the PI process as it is not required for PI activity.

GRIPP2 reporting guidelines are followed [36].

## 2 Materials and Methods

### 2.1 Types of Involvement

There were specific PI activities, and also PI in the research management activities. The specific PI activities were:

- PI Panel: Care home staff and family members (discussed in this paper).
- Activity provider facilitated resident involvement: care home residents (reported elsewhere [37]).
- Consultation events: health and care professionals, commissioners, regulators, software providers along with broader representation from family carers, care staff and care home managers (reported elsewhere [7, 8, 9]).

This paper examines the PI Panel. The PI Panel facilitated the involvement of family members, care home staff and care home managers. Five family members, three care home staff and three care home managers formed the PI Panel. Online meetings were held quarterly throughout the project. Members of the PI team chaired, facilitated and took part in these meetings. Members of the wider DACHA team informed the Panel about the research and asked questions of the panel so that the Panel influenced the detailed design, implementation and interpretation of each of the WPs.

PI activity was coordinated by four co‐applicants (including a family carer [Alzheimer's Research Network volunteer] and the Director of the National Care Forum) and a senior research associate. Members of this PI team, and another family carer, regularly attended the meetings of the core research team. Table 1 summarises the project activities and processes and the groups of people represented. In the second year of the project the family carer co‐applicant resigned from the project and a person experienced as a family carer was recruited to the PI team.

**Table 1:** Public and stakeholder roles and processes in the DACHA study.

**Represented groups**	**Study process/activity**	**Frequency**
Care home residents	Activity provider facilitated public involvement (PI)	Three care homes, eight rounds of involvement
Care staff, managers and family carers	PI Panel	One group, meeting quarterly throughout the project
Health and care professionals, commissioners, regulators, software providers	National Expert Consultation Group	Consulted three times throughout the study
Family carers, care providers, software providers, health commissioning and innovation, data governance, data policy implementation	Study Steering Committee	Six meetings over the course of the study
Chief investigator, senior administrator, senior research associates (weekly), PI Team including family carer (monthly)	Core Research Team	Weekly meetings throughout the course of the study
Co‐applicants responsible for PI (including family carer and representative of National Care Forum), senior research associate dedicated to PI activity	PI Team	Monthly meetings
Co‐applicants, research associates, senior administrator	Research Management Team	Meetings every 2 months throughout the course of the study

### 2.2 Materials for Analysing the Process and Impact of PI

Records of the PI activities included:

- Tracked actions of DACHA team members in response to input from the PI Panel through Research Management Team (RMT) minutes and questionnaire sent to DACHA team.
- Minutes of PI Panel meetings.
- Minutes of PI team meetings.

- Notes of small group reflective discussions on PI (held at RMT away day).

- Feedback from and discussion with members of the PI Panel (including the use of UK Standards for PI, and leading to a reflective article by panel members [38]).

Records of project wide activities included:

- Minutes of RMT meetings.
- Minutes of Core Research Team meetings.

### 2.3 Analysis

Analysis was iterative beginning with reflective discussion with the PI Panel in the 12th meeting, followed 3 months later by a reflective discussion by the RMT, allowing the involvement of PI Panel members and research team members in initiating analytical consideration of PI in the project. Content of the discussions were recorded in note form. These notes were then treated as part of the record of PI activities.

All these records of PI activities were read (A.K. and K.M.). The content was condensed and collated to allow triangulation between these and the records of the research project. This allowed us to explore the relationship between input from PI Panel members and decisions and activities in the conduct of the research project, to track developing impacts, identify potential missing responses to PI input and to identify themes (A.K. and K.M.). Emerging themes were discussed and developed with M.K., K.M., R.C., J.M. and then the rest of the co‐authors.

### 2.4 Recruitment

We recruited PI Panel members through the Alzheimer's Society Research Network, the National Care Forum, contacts with other care home researchers, existing university PI groups and informal networks linked to the research team. We prepared a role description identifying necessary experience: supporting family/close friend living in a care home; working as a carer or a manager in a care home; supporting a person who has dementia. Panel members would need good communication skills; the ability to participate and contribute in meetings; ability to respect other people's views and perspectives and work sensitively with people from diverse backgrounds. Remuneration was £20 per hour for 2 h of each meeting and 2 h of preparation which could be paid either as a shopping voucher, money (for which individuals would needed to register with the university to fulfil UK employment law) or care homes could invoice the university.

### 2.5 Data Recording—Records of Meeting and Content

PI panel meetings were held online using the Zoom platform. With the agreement of people attending the meeting, meetings were recorded. PI team members also took notes which, with the recordings, were used to compile draft minutes of the meeting, shared with panel members for their amendments.

## 3 Results

### 3.1 Description of Involvement Activities

The PI panel met 17 times. Total attendances were: family carers 53; care staff 23; care home managers 24; DACHA PI team 70; DACHA WP teams 36 (see Table 2). Table 3 shows the meetings, attendees, agenda items, points emerging from discussions and how these were taken into the study. Each of the five WP teams came to the panel at least twice, with three teams engaging four times with the panel. Panel members were sent an agenda and supporting information 2 weeks before each meeting. To make sure information was clear for panel members, PI team members fed back to research team members on draft information which was then edited before being sent to panel members. This helped the panel to understand the methods and purposes of each part of the study so that they could raise their questions and comments and contribute to discussions and planning. Panel meetings began in June 2020 when care home managers, staff, families and researchers were dealing with COVID‐19 with it's massive impact on care homes, care home staff and on older people. Panel meetings were chaired by members of the PI team, who opened each meeting by facilitating an icebreaker activity which encouraged all attending the meeting to introduce themselves as individuals rather than simply in terms of professional or caring roles. Meetings included presentations, small group discussions in break‐out rooms, large group discussions and use of a visual collaboration software application. Meetings lasted 2 h with a refreshment break. There were typically three main agenda items for each meeting, including the Panel process, planning each of the research activities and feedback about actions taken in relation to discussions in previous meetings. Meetings ended with an opportunity to share notable points which further allowed Panel members to shape their thinking.

**Table 2:** PI panel meetings showing dates and numbers and roles of attendees.

Panel meeting number and date	Panel members—family carers	Panel members—care staff	Panel members—care home managers	PI team members	DACHA team members
1. 30/06/2020	3	0	4	4	1
2. 11/09/2020	3	2	3	5	1
3. 05/02/2021	4	1	2	6	1
4. 07/05/2021	3	3	3	6	3
5. 06/08/2021	5	1	2	6	2
6. 15/10/2021	4	2	0	5	4
7. 05/11/2021	3	1	1	5	0
8. 04/02/2022	3	1	1	4	4
9. 06/05/2022	4	3	1	5	3
10. 05/08/2022	2	2	0	4	2
11. 04/11/2022	5	1	1	3	7
12. 03/02/2023	3	1	1	4	1
13. 05/05/2023	3	1	1	3	1
14. 29/06/2023 meeting stopped through ill health					
15. 04/08/2023	1	1	1	3	3
16. 03/11/2023	3	2	1	4	1
17. 01/03/2024	4	1	2	3	2

**Table 3:** Table of PI panel meeting agenda, key points and actions.

Panel meeting number and date	Agenda items	Key points emerging	How key points taken account of in project
1. 30/06/2020	Introduction to DACHA project	Will the two resident public involvement care home groups in Norfolk be representative?	
	Information about how the panel will work	When will the panel get information to look at so they can feedback on it?	Share research plan with key milestones for the project with the panel members.
	Work package 2— repository of data, is it reasonable to reuse original participants' data?	Panel members in favour of reusing data. Advised raising with ethics committee for advice.	Advice sought from ethics committee that originally approved a trial included in the repository. The view of PI panel that panel members were in favour of reusing data was shared with ethics committee. This trial is now included in the Trial repository.
	Information on reimbursement for PI panel membership		
2. 11/09/2020	Work package 1, review 1, emerging findings about outcome measures, including InterRAI	Few examples of outcome measures which incorporate representation from families.	The key points informed the interpretation of findings and the discussion in the paper reporting the literature review of outcome measures used in care home research, in which the following points were made:
		InterRai as a long list, there should be attention on how the factors interact for individuals.	
	Work package 1, review 2	Categories broad and may not pick up on nuance, e.g., for a person quite ill with dementia.	(1) Outcome measures that are used in research are not often used in the day‐to‐day life of care homes. (2) common research outcome measures, specifically Barthel Index, were viewed as outdated as care homes often routinely collect a wider range of data about residents. (3) residents can have day‐to‐day fluctuations in outcomes, and most research tools only measure outcomes at a single time point so may not collect an accurate picture of residents.
		The functional implications should be emphasised—e.g., potential for social isolation if sensory needs not met.	
		Much of this information currently already collected, but time consuming on paper, not all easily shared with families, and not always used to support responsiveness to change in resident condition.	
		For SK to link with software providers.	
		DACHA has useful role in helping establish purpose of an MDS. This could help consistent information be collected across care homes.	DACHA team wrote and published ‘Developing a minimum data set for older adult care homes in the UK: exploring the concept and defining early core principles’.
3. 05/02/2021	Terms of Refence and Agreed ways of working	Agreed.	DACHA team wrote and published ‘Developing a minimum data set for older adult care homes in the UK: exploring the concept and defining early core principles’.
	DACHA project and purpose of an MDS, in context of other practice initiatives	Need for an MDS to capture individual functional needs, not simply scales.	
		That PI can contribute voices of residents, family carers and care home staff to development of MDS.	
		An MDS must replace other data recording, not add to it—ask care home managers what they collect regularly, what is used.	
		To have value MDS must provide feedback to care home managers.	
		Algorithms to flag, e.g., deterioration would be valuable.	
		Data must be held securely.	
		MDS should include personal preferences.	
		MDS needs to be easily accessible, used, with staff trained in using it, may need to have requirement for regular data entry.	
		Critical for an MDS to have integration with NHS data.	
		Transferrable between care homes if a person moves care home.	
4. 07/05/2021	WP3 findings from realist review of uses of minimum data sets	Importance of frequent data entry for best use of MDS in supporting care—staff need understanding to have ownership.	Impact funding sought to develop accessible messages to care staff about principles of MDS and their key role.
	WP1 literature review 1, to inform panel how their input informed the review, and the results	Ideally MDS should facilitate two way communication between care home and family.	Informed thinking about whether outcome measures used in research measure what they aim to measure and whether they measure what is most important to residents, family and friends and staff.
		MDS should enable efficient responses for care homes to requests for data.	
	List of types of data collected currently in care homes compiled by panel members— discussion of utility/what would be useful to collect	MDS should give a real sense of the whole person, incorporating wellbeing and mental health as well as physical health.	Influenced reporting in the paper reporting the literature review, measures used in research are frequently not relevant to everyday life in care homes, and don't take account of wellbeing.
		MDS should facilitate resident involvement in data collection.	
	Work package 3, the survey of care home managers about data collected	Barthel scale is physically focussed, insensitive to change, seen as outdated, but is used sometimes in care homes to calculate staffing needs.	Development of an infographic to share this message widely to increase general understanding.
		Of the 400 tools used in research, only MMSE and Barthel recognised by panel members.	Survey questions informed by list drawn up by panel members.
		Reflection on the extensive demands on care homes to share information, with much duplication.	
		Examples of information manager chooses to collect to help monitor individual wellbeing and care provision.	
		Examples of data provided to CQC but no feedback on performance compared to other homes.	Questions added addressing wellbeing and mental health. Increased emphasis on mood and perspectives of relatives in the survey.
		Encourage responses from direct care staff.	Draft of survey shared with panel members for further comment.
		Incorporate questions about data for wellbeing and mental health.	
		Incentivise completion—e.g., offer training/information back from university for care staff.	
5. 06/08/2021	Open item—what panel members think DACHA should be considering in relation to resident's information	Recording information about diversity of residents, including, e.g., ethnicity and sexual orientation, so that outcomes for different groups can be seen.	
		MDS needs to be able to develop over time.	Amendments made to infographic which was then shared via DACHA website.
	Infographic to show data sharing issues—to communicate widely the insights from previous panel discussions (see meeting 4)	Sensitive prompts to consider detailed unmet needs.	These points were incorporated into the activity pack used to facilitate resident public involvement.
		Prompts for identifying change in resident's condition.	Panel discussion prompted discussion in the project team about who's perspectives are incorporated in an MDS; resident, relative, provider, other?
	Developing the public involvement with care home residents	Prompts for contact with family.	
	Feedback to Panel about changes made to Work Package 3 staff survey in light of their input	Should represent two direction information flow.	
		CQC and safeguarding should be included.	This separate project team contacted panel members.
		Reduce text in infographic.	
		Provide brief information for involved residents of information stored about them, as context for discussion.	
	Invitation to additional meeting with DACHA team member LI to inform development of a study to find out residents' and relatives' priorities for research using trials archive	Use pictures and short video as well as text.	
		Be aware of communication needs of involved residents.	
		Use photo library from Centre for Ageing Better.	
	Invitation to join DACHA study Facebook	Panel members happy to be emailed about this project.	
	Invitation to contribute to a project about data sharing, care homes and GP practices		
6. 15/10/2021	WP4 workshop re: data linkage	Data collection already happening in care homes. Quality of data collected will depend on whether it has value to the right people.	WP4 shared learning with rest of DACHA team (esp. WP5).
	Update about PI with care home residents (first round of activities in with two APs)	Need to identify who has access to the data being collected. How do we ensure it's being used to improve resident care. Also keen for residents and family carers to be able to access collected data.	WP4 decided to return to ensure PI were involved when developing their analysis plan.
		Care home staff want to know more about hospital admissions and how to balance min and max data sets.	A ‘next step’ documented in the minutes was to explore option of providing a plain English privacy statement on the DACHA website (but unclear if actioned).
		Data safety is paramount. Panel members happy with WP4's plans re: data safety.	Could be answered via BH's survey.
		Questions about how many care homes are using digital systems.	
		Need to explore ways to make sure people living with dementia are included (and to consider how other projects have done this).	PI team incorporated feedback from APs and put in options to help involved people LWD (e.g., a range of activities to choose from, prompt cards, flexibility in how activities were run).
		To consult family members with PoA.	
		Positively received and panel members happy care homes were tailoring participation to individuals' wishes.	
7. 05/11/2021	Feedback from DACHA survey re: findings (what data care homes are collecting, perspective on data sharing and an MDS)	Questions about the care homes that participated (how representative they were of overall). Keen for more information re: analysis and context (e.g., to get insights on why some responding homes did not collect NHS numbers). Some surprises at what some care homes do not collect and how this may impact on care. Questions about how complete the care homes' data is.	Consideration of how to present survey findings in a nuanced way.
	DHSC provider data set	Feeling that survey focused more on resident health than wellbeing. Quality of life is missing.	Quality of life measures to be included in the MDS.
	Other	Widespread use of digital technology surprising.	Promoting this message, e.g., through the infographic and public resources/plain English summaries.
		Important that data sharing is two‐way. Need for context with data, staff training, supportive use of league tables.	
		MH positive re: contacts made between DACHA and Skills for Care Workforce Intelligence, with member of SFC joining the DACHA steering committee.	
8. 04/02/2022	WP5 overview and discussion of study recruitment	Information sheet—difficulty of balancing ethics committee's need for technical language and residents still being able to understand it. Suggested amendments, e.g., to add how long data would be held. Importance of having different options, e.g., easy read.	Recruitment materials amended (information sheets made clearer, flowcharts added), easy read options included.
	Data items in the MDS (which outcome measures best capture wellbeing and quality of life)	Looked at ASCOT, QUALIDEM and ICECAP‐O. Liked ASCOT but?missing sense of overall wellbeing, liked QUALIDEM but is long. Need to consider (a) the types of care homes taking part (and if findings generalisable) and (b) that staff may rate QoL more highly than a family member.	Incorporation of feedback into consultation re: measuring QoL. Discussion of how SWAP can explore some of issues mentioned.
	Update re: Study Within A Project (SWAP) about domiciliary care, offer to be involved in PI for this	Residential care homes and family members should be involved in data interpretation.	WP5 said would try to return for this. Also to share benchmarked data with care homes in their area.
		Members of the panel volunteered to be involved.	Some panel members joined the Study Within A Project (SWAP).
9. 06/05/2022	DACHA consultations	Panel asked to help trial consultation survey.	Feedback incorporated into survey design.
	WP4: Learning and actions from last panel	Keen for two‐way flow of information will relatives but acknowledge goes beyond DACHA's remit. However could be a recommendation (future‐proofing).	This recommendation has been communicated at conferences and in other outputs.
			Feedback about trusted data sources taken into account by WP4. Inclusion of community service utilisation re‐ranked as high priority for MDS capture.
			WP4 ensured community health records linked in Pilot MDS. Community health data collected and analysed in pilot.
	WP4: Current challenges—data sharing agreements	Discussion re: trusted data sources. Keen for inclusion of district nursing records.	Panel thanked for their help, still able to feedback via email if desired.
	Infographic feedback	Access to information from GPs is problematic for care homes.	Infographic uploaded to website.
10. 05/08/2022	WP5: update and discussion (how to engage with residents and families about DACHA, maintaining care home engagement over time)	Discussion of strategies, e.g., open days, posters, linking recruitment to monthly resident reviews, use of newsletters to keep engaged.	WP5 team used a newsletter to keep in touch with care homes.
	Update of PI activities with residents	Panel advised on how to frame exploring QoL.	Advice taken into account when designing activity pack.
	Facebook relaunch	Panel asked to look at Facebook page and feedback. Some mixed feelings about how useful/appropriate it would be.	Facebook page amended but eventually taken down.
11. 04/11/2022	WP4: Analysis protocol (what is already known, what would be useful to know)	Interest in use of data for constructive benchmarking, outcomes that would be interesting (e.g., pressure ulcers, UTIs), importance of context with data—e.g., the particular population of any one care home, staff skill mix and level of training. Need to think about who is receiving the data and their understanding of it. Pros and cons of benchmarking.	WP4 mindful of feedback when completing their analysis.
	Feedback from QoL consultation	Panel fed back about how they would like to learn about the findings (infographic or slides, inclusion of more detail).	QoL consultation is summarised in a report of the national consultations on the DACHA website.
	WP5: Recruitment challenges	Discussion of how to maximise recruitment via family members (when residents cannot consent) as relatives are asking what data would be taken, why DACHA needs it, how their relative would benefit from taking part. Panel suggest making sure activity coordinators have information to pass to relatives, assurance of confidentiality, information meeting for relatives led by care home manager, importance of personal contact from a researcher.	Researcher reflected on the feedback which reinforced the range of approaches being used.
12. 03/02/2023	Implementation	Discussed factors that would potentially affect implementation. Importance of speaking to managers and care staff, also considering the MDS with respect to the ‘bigger picture’.	Reinforces decision for WP5/implementation team to be interviewing and gathering feedback from care homes.
	Reflective exercise	Discussed the panel's experiences of being part of DACHA and any feedback.	Added action points to panel minutes. Keen to see a paper and/or report about how the panel has influenced DACHA (A.K. focusing paper on this, K.M. gathering impact data). Started having brief bullet point updates about ongoing work packages at start of each panel (to help panel members to keep track over time).
13. 05/05/2023	WP5: Update and MDS preview	Discussed potential differences in people in different staff roles completing outcome measures on behalf of residents.	Question asking about job roles added to interviews about how care staff complete outcome measures (this fed back to PI panel in meeting 15).
	WP1: Discussion of findings of review 2	Discussed difficulties of participating care homes completing all data entry in one locality involved in the study and how best to address this. Concern raised by panel of impact on care homes of pressure to complete data entry for the DACHA pilot.	WP5 team followed suggestions of the panel in resolving difficulties of data entry completion (offered online debriefs with managers, sent email to managers apologising for issues, made a roadmap for managers, newsletter to update care homes, offered contact via email and phone) (feedback to panel on this in meeting 15).
	Discussed contributing to writing activities	Discussed wastefulness re: number of outcome measures used in research, how it is unclear how or why they are selected for use. The panel expressed an interest in any end of life measures included.	Comments included in plain English version of the review and shared with lead reviewer. Attempted to publish the plain English version but was not picked up—put on website.
		Several panel members expressed an interest.	M.K. and E.A. wrote paper, published with K.M. Panel members invited to contribute to write up of Panel public involvement in the DACHA study.
14. 29/06/2023	Panel not completed—illness of panel member.		
15. 04/08/2023	WP4: Analysis update	The panel asked questions and discussed what they would be interested in learning from the data (such as how medication is used) and the complexities of interpreting data (e.g., frailty scores). Panel interested in being involved in interpretation of the data.	Feedback incorporated into analysis plan. WP4 event (March 2024) re: data analysis and interpretation.
		Panel asked if data would be collected on DNAR and on end of life plan (highlighting the difference between these).	WP4 checked if this could be added to GP data request. Information on discussion of preferred place of death was obtained from community. services data set in the pilot MDS, and discussed in publication of the analysis of pilot data.
	WP5: Feedback from PI team about how feedback on care home pressures was actioned (see meeting 13).	Panel emphasise importance of staff being able to use the information in an MDS to improve care for individuals.	
		The panel were pleased that this had been actioned.	Impact funding to develop accessible information for care staff about their critical role in data (entering and use of data).
16. 03/11/2023	Suggestions to enhance care home recruitment to SWAP	The panel made several suggestions, e.g., posters up in staff rooms with offer of voucher, easy link to make contact and one to one interviews rather than focus group.	This approach was used by the SWAP and also when recruiting care home staff for the third DACHA consultation.
	WP2: Update on VICHTA follow‐on study	The panel felt researchers should be able to submit questions. Discussion of potential uses of VICHTA data.	VICHTA researcher to offer panel members chance to respond to VICHTA consultation when live, summer 2024.
	Discussion of an additional panel meeting	The panel expressed interest in a final event in Spring 2024. The panel reflected on how members' involvement in, or understanding of, research has increased since becoming involved in DACHA.	A panel event was arranged for Spring 2024. Panel reviewed and comments on PI section of final report.
17. 01/03/2024	Reflection on participation in DACHA PI panel		
	Emerging findings from WP5, pilot of MDS in care homes	Discussion on how the QoL measures performed in the pilot, how the information might be used to inform care, tensions between standardisation versus tailoring of e‐records software for care homes.	Summary of impact of PI on DACHA study sent out to PI panel members.
	Feedback from principle investigator of DACHA on impact of PI	Discussion on how answering QoL questions had changed care staffs' perceptions of what was important to individuals.	

GRIPP 2 Long Form for reporting public involvement [39]		

Section and topic	Item	Reported on page no
Section 1: Abstract of paper		
1a: Aim	Report the aim of the study	1
1b: Methods	Describe the methods used by which patients and the public were involved	1
1c: Results	Report the impacts and outcomes of PPI in the study	1
1d: Conclusions	Summarise the main conclusions of the study	1
1e: Keywords	Include PPI, ‘patient and public involvement’, or alternative terms as keywords	1
Section 2: Background to paper		
2a: Definition	Report the definition of PPI used in the study and how it links to comparable studies	2–3
2b: Theoretical underpinnings	Report the theoretical rationale and any theoretical influences relating to PPI in the study	2–3
2c: Concepts and theory development	Report any conceptual models or influences used in the study	2–3
Section 3: Aims of paper		
3: Aim	Report the aim of the study	2
Section 4: Methods of paper		
4a: Design	Provide a clear description of methods by which patients and the public were involved	4–6
4b: People involved	Provide a description of patients, carers and the public involved with the PPI activity in the study	4–6
4c: Stages of involvement	Report on how PPI is used at different stages of the study	4–6
4d: Level or nature of involvement	Report the level or nature of PPI used at various stages of the study	4–6
Section 5: Capture or measurement of PPI impact		6–12
5a: Qualitative evidence of impact	If applicable, report the methods used to qualitatively explore the impact of PPI in the study	6–12
5b: Quantitative evidence of impact	If applicable, report the methods used to quantitatively measure or assess the impact of PPI	NA
5c: Robustness of measure	If applicable, report the rigour of the method used to capture or measure the impact of PPI	NA
Section 6: Economic assessment		
6: Economic assessment	If applicable, report the method used for an economic assessment of PPI	NA
Section 7: Study results		
7a: Outcomes of PPI	Report the results of PPI in the study, including both positive and negative outcomes	6–12
7b: Impacts of PPI	Report the positive and negative impacts that PPI has had on the research, the individuals involved (including patients and researchers), and wider impacts	6–12
7c: Context of PPI	Report the influence of any contextual factors that enabled or hindered the process or impact of PPI	6–12
7d: Process of PPI	Report the influence of any process factors, that enabled or hindered the impact of PPI	6–12
7ei: Theory development	Report any conceptual or theoretical development in PPI that have emerged	13
7eii: Theory development	Report evaluation of theoretical models, if any	13
7f: Measurement	If applicable, report all aspects of instrument development and testing (e.g., validity, reliability, feasibility, acceptability, responsiveness, interpretability, appropriateness, precision)	NA
7g: Economic assessment	Report any information on the costs or the benefit of PPI	NA
Section 8: Discussion and conclusions		
8a: Outcomes	Comment on how PPI influenced the study overall. Describe positive and negative effects	6–12
8b: Impacts	Comment on the different impacts of PPI identified in this study and how they contribute to new knowledge	6–12
8c: Definition	Comment on the definition of PPI used (reported in the Background section) and whether or not you would suggest any changes	2–3
8d: Theoretical underpinnings	Comment on any way your study adds to the theoretical development of PPI	13
8e: Context	Comment on how context factors influenced PPI in the study	6–12
8f: Process	Comment on how process factors influenced PPI in the study	6–12
8g: Measurement and capture of PPI impact	If applicable, comment on how well PPI impact was evaluated or measured in the study	NA
8h: Economic assessment	If applicable, discuss any aspects of the economic cost or benefit of PPI, particularly any suggestions for future economic modelling.	NA
8i: Reflections/critical perspective	Comment critically on the study, reflecting on the things that went well and those that did not, so that others can learn from this study	6–13

Abbreviation: PPI = patient and public involvement.

### 3.2 How the Involvement Influenced the DACHA Project

**Figure 2:**
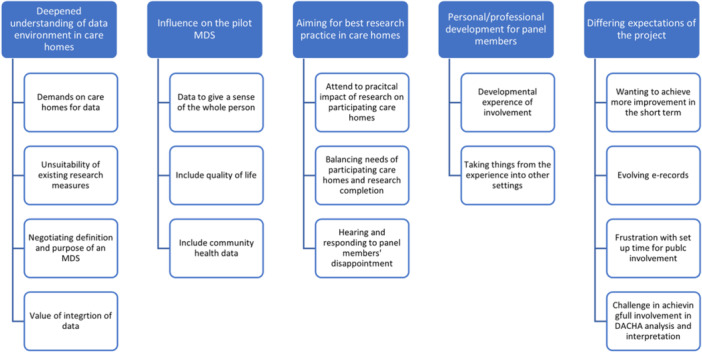
Results: Themes and subthemes.

Influences of the involvement were extensive, pervasive and dynamic, as researchers' appreciation of the care home data context deepened, PI contributors developed understanding of research approaches and influence early on in the project had ongoing effects later. Themes are listed, illustrated with subthemes (Figure 2) and discussed below.

### 3.3 Themes

1. *Deepened understanding of the data environment in care homes.*
2. *Influence on the pilot MDS.*
3. *Aiming for best research practices with care homes.*
4. *Personal/professional development for PI members.*
5. *Expectations of the project.*

#### 3.3.1 Deepened Understanding of the Data Environment in Care Homes

Data that care homes were expected to provide to other organisations with little feedback, was a key topic regularly discussed in the PI panel in varied contexts. Discussions were iterative, as researchers engaged with input from the panel, came back to the panel to report how they had responded to that input, and discussed implications for the next stage of work.

A literature review of measures or instruments used in care home research was conducted [4] to discover if any of the measures would be useful as part of an MDS. The emerging findings of this review were discussed with the PI panel, with family members, care staff and managers all arguing that existing measures gave insufficient attention to mental health or wellbeing. They emphasised the importance of sensitivity to change over time, with different aspects of needs coming to the fore at different times in the trajectory of a person's stay in a care home. Discussion with the panel members drew attention to how little these measures used in research included family representation, an important source of information about residents. Existing measures were thought to be insensitive, lacking the detail and range of information now routinely collected by care homes to inform their care of residents. Care staff illuminated the current usual practice of monthly wellbeing reviews, although not necessarily shared with families, as family members concurred. The panel discussion was reflected in the literature review report, in particular that there is little relationship between outcome measures used in research and routine data recording in care homes, research measures appear outdated in relation to information recorded in care homes and insensitive to day‐to‐day fluctuations for residents [4].

As researchers and panel members listened to each other and worked to reach shared understandings about the purpose of a MDS in care homes, researchers found it difficult to explain the parameters for data with potential for inclusion in an MDS. The Panel's thoughtful questioning and challenging discussions prompted the DACHA team to negotiate, agree and propose a definition of, and purpose for, an MDS [3].

PI Panel members emphasised the desirability of integration between care home records and NHS data, transferability for a person moving from one care home to another and usability for staff. Detailed accounts of the day‐to‐day realities of dealing with data in care homes were crucial to the research team appreciating the complexities of the demands placed on care home staff to provide data to other agencies. Very similar data is required by many different stakeholders, and different departments in the same stakeholders, in different formats, leading to duplication of effort. The experience for care homes is of providing data but getting nothing back in the way of analysis or feedback on how their outcomes relate to those of other care homes. Three members of the panel (two managers and one senior carer) compiled a list of all the types of data that they recorded regularly and this was used to help design a survey sent out to care home staff [6]. The Panel helped design an infographic to communicate the data demands on care homes [40].

It was clear that an MDS should draw on data already recorded and not add to the burden of care homes. To add value, and therefore to motivate implementation, an MDS must provide feedback to care home managers on the performance of their care home, to feedback to teams and drive improvement. The PI Panel was aware of the importance of the work and increasing national focus on care homes and data as a consequence of the impact of the COVID‐19 pandemic and policy responses.

#### 3.3.2 Influence on the Pilot MDS

##### 3.3.2.1 Quality of Life

The family members, staff and managers in the PI Panel were clear that an MDS should give a sense of the whole person, incorporating wellbeing and mental health. Having fed into the development of an online survey for care home staff, the results from the survey were fed back to the panel and discussed to inform the analysis. This drew attention to the dearth of information being recorded in practice about quality of life. Further analysis revealed that information about quality of life was recorded by fewer than a third of respondents to the survey [6]. The impetus from the PI Panel was to push the developing DACHA MDS beyond international examples to incorporate quality of life. DACHA researchers and PI Panel members discussed the pros and cons of quality‐of‐life measures that could be part of an MDS. The PI Panel helped develop activities to facilitate PI of care home residents about quality of life [37], and their views also informed the development of a consultation with wider stakeholders on their utility and usability [8]. The preferred measures were included in the pilot MDS that was trialled in 45 care homes with 996 residents [10]. Three of the four measures piloted were found to have acceptable psychometric properties [41] and used to better understand the factors associated with different constructs of residents' QoL (e.g., emergency hospital admissions) [42].

##### 3.3.2.2 Community Health Data

DACHA researchers from THF brought expertise in data linkage and analysis of sets of routine health data. While simple in conception, the execution is complex both technically and in relation to governance processes. Health services for people in care homes in England are provided by the NHS and by General Practitioners (GPs). The NHS is made up of Trusts, organisations with a geographical and functional focus (e.g., acute care) and with their own governance arrangements. THF DACHA team members drafted accessible presentations, discussed and refined these in collaboration with DACHA PI research team members and met the PI Panel four times (Panel meetings 6, 9, 11, 15). PI Panel members were able to develop an understanding of a technically complex research approach so that they could contribute their views and get feedback on how their input had influenced the research. The PI Panel was excited by the potential of linking individual care and health records, and particularly emphasised the value of linking information on community health care including district nursing and community rehabilitation services. Panel members working in care homes commented on the strength of district nursing records as a source of reliable information about residents' health and input from health services. This resonated with PI with care home residents [37] who put priority on better information about their appointments with health professionals. When the DACHA RMT agreed principles and priority information for a MDS, PI team members advocated for community health information to be included in the pilot MDS through data linkage, as this was prioritised by PI Panel members and residents. In response the pilot MDS collected the mean number of community service appointments overall, and broken down for speech and language therapy, continence, district nursing, podiatry and community rehabilitation. All were reported over 1 year and could be summarised across different subgroups, for example by resident or care home characteristics, to understand variation.

#### 3.3.3 Aiming for Best Research Practices With Care Homes

The involvement of a family carer and a director of the NCF from the early stages of project development and writing the funding bid helped to ensure that plans considered the practical implications of carrying out the research for care homes and for the people living in them. The DACHA project aimed to create new ways of working and doing research in and with care homes, so that the outputs benefit not only researchers but also residents. The research team's awareness of the demands on care homes and the workload for staff and managers was sharpened by the Panel discussions over the project. Participating in research would bring demands over and above day‐to‐day practice, which was still recovering from the impact of COVID‐19, and the time needed for participation should be made clear up‐front to care homes, as transparency aids the homes' planning and commitment. There was a sense that the needs of the care home should be central and the demands of taking part in the research should work around this. A critical point in the timings of the research project challenged this value, leading to difficult discussions both in the PI Panel and the DACHA team. The pilot of the MDS involved care homes completing additional measures at two time points for each participating resident in their home, in addition to the extraction of routinely collected data for these residents. There was a deadline for completing the measures in order for the e‐record software providers to extract the data. There was a miscommunication, some care home managers were not informed that there was a request for additional measures to be completed and a deadline for these.

The researcher dealing with the consequences of the missed information was balancing the evident stress of care home managers and staff when approached to complete data entry in a short space of time, with the demands for the viability of key aspects of the research study. When the researcher presented this to the PI Panel as part of an update on the progress of the study, Panel members expressed disappointment that, despite their involvement and contributions throughout the study and the expressed wish of the study to work well with care homes, past poor practices experienced by some panel members in other research projects had been repeated. The respectful but challenging exchange in the PI Panel meeting, which a care home manager panel member commented benefitted from experienced chairing to ensure all views were heard, respected and understood, did develop an action plan to mitigate the impact on the care homes involved. This included offering online debriefing sessions to care home managers, a communication from the study lead apologising for the issues, and the offer of contact for care home managers with researchers by email or phone. A ‘road map’ of future dates was suggested by a care home manager at a participants' debriefing session and sent to all participating care homes.

Most of the affected care homes strove to complete the additional measures in the short time frame believing that the project will benefit residents in the long‐term. Others, already stressed by issues other than the research, withdrew from the study. A care home manager Panel member reflected on the importance of endings and the impact on any future research participation for these homes. The local research nurse contacted the withdrawn care homes to understand learning and keep communication channels open for future research.

The researcher had an uncomfortable position at the PI Panel as the spokesperson for the conduct of the fieldwork, as was discussed at an RMT meeting. For some team members the practical problems faced by the project were seen to make unavoidable any additional pressure put on participating care homes. Indeed this was also respecting the individual residents in those participating homes who had given consent for their records to be used in the study. Others argued that it was important for the project to act consistently with the value of giving care homes a voice in the research both through the PI Panel and relations with participating care homes.

Even though we aimed to ensure that the needs of the care home were central and we had strong PI, undue pressure on care homes can quickly arise (e.g., through a miscommunication).

#### 3.3.4 Personal/Professional Development for PI Members

Although it was not an explicit aim that involvement in the Panel be developmental for the members it was clear from a reflective session held in meeting 12 that it was for some. Panel members described their motivation for getting involved as wanting to make a contribution. Family carers felt they could draw on their experience of having their spouse or parent living in a care home. They were also drawing on other life experiences including work or volunteering in health or care‐related settings. Some family carer Panel members thought they should have less influence than researchers and people working in care homes. One family carer said they had learned from the project, and subsequently raised awareness of PI while volunteering with dementia groups.

By this 12th meeting, Panel members were able to describe initial concerns that taking part in the Panel would raise uncomfortable differences in perspectives. One family carer described being fearful they would be ‘too negative’ because they had not been able to find good care for their relative. This person worked in an organisation aiming to support and promote social care so was wary of the potential conflict with that role if they spoke negatively about care, but reflected that taking part had sparked ideas for improvement of her organisation's work. A care staff panel member described their initial caution in contributing because of the ‘disconnect and lack of understanding’ in society of the work of social care. This person was concerned that the research plan would be unrealistic. However, they described the ‘morale boost’ of the unexpected opportunity to connect with a group of people who, from different perspectives, cared deeply about the subject. They had considered things that wouldn't have occurred to them, which had informed and improved their work as a carer. Care home managers in the panel valued hearing the thoughts and reflections of family members who could be frank and open in this different context, and this influenced their practice. Two members of the panel published an article, aimed at care home staff, about their experience on the panel, and the career opportunities this led to, which including as a research co‐applicant, working for NIHR [38] and a career path blending practice, research and implementation.

#### 3.3.5 Differing Expectations of the Project

Some issues of great importance to PI Panel members were beyond the scope of the project. For example, Panel members wanted the pilot MDS to share real‐time data with families. Family members and care staff were cognizant of the huge potential of linkage of individual care and health records for effective care. The scope of the pilot MDS was to create a pseudonymised linked data set that could be a proof‐of‐concept but would not identify individual residents or be directly accessible to CH staff, residents or carers (so therefore couldn't share any data with families). With the concept tested, the project would make recommendations to policy makers who were considering implementation of an MDS for care homes. Family members stressed the potential to use software to easily find out how their relative was, what they have been doing during the day, without calling care staff away from their work. Digital care planning systems were evolving and their use became more widespread in care homes during the course of the project. This functionality of family access that panel members thought important in an MDS was becoming available in e‐records systems. This information from digital care planning systems is, however, not yet linked to records held about individuals in the health system.

Some PI contributors were frustrated about the pace of setting up PI activities, timing and format of reporting meetings back to panel members and limitations of meeting on‐line rather than face‐to‐face. The Principal Investigator (C.G.) and research team responded with telephone conversations and in‐person meetings with concerned individuals and agreed action plans to try to resolve issues. The original family member co‐applicant chose to leave the project, feeling their time could be better used elsewhere.

The PI team aspired for more PI Panel involvement in data analysis and interpretation than was achieved as the complexities of study recruitment, data governance between organisations for data linkage and data extraction limited time available for analysis. Panel members were interested to contribute to writing up the work of the panel and have contributed to this paper. PI contributors reflected on the PI process and these reflections formed part of the analysis for this paper. Four members of the PI Panel (E.A., M.F., M.K. and J.W.) chose to be co‐authors of this paper, edited the early draft of the paper and contributed to findings development.

## 4 Discussion

We aimed with this paper to analyse the involvement of care home staff and family members in the context of the overall project [20, 22, 43]. The DACHA project aimed to keep the concerns and priorities of people living and working in care homes informing all stages of the project and we argue that this was achieved, as shown in the inclusion in the MDS of content prioritised by the PI Panel. We aimed to use a democratic approach, valuing different knowledges through the involvement of public contributors in decision making [23]. There is evidence in reflections from the panel that members did feel that their knowledge and experience were valued and heard. The input of the panel directly informed decisions, notably to include quality of life measures in the pilot MDS, and to link to data on community health services. The Panel was involved in implementing these decisions in the ongoing research by reviewing and giving feedback to decide which measures of quality of life should be used. There was increased work for researchers who needed to identify the data owners for community services and negotiate data‐sharing agreements. Decision making in the project was progressive. Final decisions about which items to include in the MDS were ultimately taken by the research teams working on the relevant WPs, in negotiation with the RMT, in which PI team members advocated for the PI panel perspective. Decision making was therefore a process over time rather than a single event, but we argue a shift in the ‘soft’ norms, codes and values of research practice [22] was achieved, with researchers responding to and feeling accountable to PI members.

We aimed to keep these groups' priorities central to our research, in the belief that for an MDS to be useful to improve care and usable in day‐to‐day practice, the research approach should be of close collaboration with those groups most affected who would bring unique insights. Social care has not had the research attention that health has enjoyed. Research questions and policies for practice tend to be heavily influenced by health practice needs. Care practitioners are not traditionally represented in academia. The impact of Covid‐19 further illustrated the policy neglect of the care sector [44], and strengthened our conviction for collaborative work. Researchers need to consider the political context of their research field and project [45] and relative power positions of the groups who have a legitimate interest in the research.

A democratic PI approach is fragile and can be challenged by the contractual obligations of completing a funded research project in the context of unexpected events [43]. Fragile democratic relations between the research team and the PI panel members could be argued to have been bolstered in this project by the consistent process of quarterly meetings, and by chairing that aimed to developed shared understanding and trust between Panel members and researchers attending each panel meeting. The views of the Panel were ascribed power through technical tools of agendas and reporting at both core team meetings and RMT meetings [46]. Panel members could invoke the power of authentic accounts of lived experience, but this was at times contested by researchers citing their own experience in care homes [47, 48]. Family members frustrated with the PI arrangements drew on existing social relationships to escalate these concerns, leading to feelings of vulnerability for some researchers. Social relations in PI activity are both the means for constructing shared understandings but also likely sites for misunderstanding, disappointed expectations and tension [49]. These tensions can be productive [50] but there needs to be attention to those in less powerful positions, both PI members and researchers [51].

One alternative approach to PI in this project, suggested in RMT reflections, would have been to have PI representatives linking to each WP team rather than a central Panel. This may have strengthened social relations between researchers and PI members, increased the PI members' opportunity to understand detailed aspects of the research methods and help to resolve challenges. However, this would have made it more difficult for PI to hold the overall project to account on issues such as the focus of the project including the mental health and wellbeing of people living in care homes. An ideal might be to combine both approaches, although this would need a greater time commitment from PI Panel members and increased support to negotiate the wider role.

This PI Panel brought together in the same meetings care staff, care home managers and family members of people living in care homes. There were some difficulties in voicing views and experiences openly in this mixed group at first. We made use of the function to have the individual groups meeting in smaller ‘breakout’ groups within the online meetings. As the project progressed however trust built between group members, and members valued hearing alternative perspectives. As described earlier in the paper, people living in care homes had PI facilitated by care home activity staff [37]. Further development of the PI Panel approach should consider supporting direct representation in the Panel from people living in care homes [52].

Social relations were more complex than simply between PI panel members and the research team. Many individuals had more than one role or identity [47]. The research team included members linked to practice (NCF) and with family carer experience. Researchers also have relevant family or social care practice experience. The research team grew substantially to conduct the study. Some of the team know each other well while others were working together for the first time. PI Panel members, both family members and practitioners, brought experience in other roles including research, social care practice, advocacy and volunteering [53]. These multiple roles and identities also underpin complex power relations [22, 54]. Reflections in the RMT meeting and in the PI Panel touched on these issues. Notably the concern and sense of responsibility expressed by PI Panel members either initially sceptical that a research project could be ‘realistic’, or struggling to reconcile a sense of responsibility to advocate for the care sector with personal experience of poor practice. Some PI members moved into other research related roles, meeting the policy agenda for developing social care research capacity [55]. These overlapping roles brought shared experience and empathy to the panel interactions, but also, with individuals as ‘boundary spanners’, the potential for advocating for social care research grounded in practice.

We argue there have been ‘soft’ effects from the PI process that are important for growing a social care research practice. Researchers' understanding of, and attitudes towards, residents and care home staff have benefitted from a deeper appreciation their situation. Many of us have completed the project having learned far more than can be wrapped up and capitalised on in this project and the challenge is to sustain and re‐invest this learning. This project had resource for PI co‐applicants to contribute to project design and committed resources to support PI throughout. While this is recommended good practice [56] it is not easy to identify funding for research development. For PI to be influential from the earliest stages of project inception it is necessary to support and sustain interest groups of, for example, care home staff and people living in care homes, beyond individual projects.

## 5 Conclusion

The DACHA project shows that with integrated PI, commitment demonstrated from the Chief Investigator and sufficient resources designed into the project, collaborative research leading to outcomes prioritised by those most affected can be achieved. The PI not only informed the MDS but also deepened our understanding of the context in which we were working and provided both accountability and support when there were issues. The PI exemplified a ‘social practice of dialogue and learning between researchers and the public’ [22]. The next steps of development and implementation of an MDS for care homes should build on this example of socially transformative PI practice [24], incorporating relevant knowledge and experience, to minimise negative unforeseen consequences. There is a need to tap the deep knowledge in practice by spanning boundaries between research and practice, and rapidly enhance practitioner research in social care.

## Author Contributions

**Anne Killett:** conceptualisation, investigation, funding acquisition, writing–original draft, writing–review and editing, project administration. **Kerry Micklewright:** conceptualisation, investigation, writing–review and editing, formal analysis, project administration. **Rachael Carroll:** conceptualisation, investigation, writing–review and editing. **Gizdem Akdur:** writing–review and editing, project administration. **Emily Allinson:** writing–review and editing, investigation. **Liz Crellin:** investigation, writing–review and editing, formal analysis. **Kaat de Corte:** investigation, writing–review and editing. **Margaret Fox:** investigation, writing–review and editing. **Barbara Hanratty:** investigation, funding acquisition, writing–review and editing. **Lisa Irvine:** investigation, funding acquisition, writing–review and editing, project administration. **Liz Jones:** conceptualisation, investigation, funding acquisition, writing–review and editing. **Marlene Kelly:** investigation, writing–review and editing. **Therese Lloyd:** investigation, writing–review and editing. **Julienne Meyer:** conceptualisation, investigation, funding acquisition, writing–review and editing. **Karen Spilsbury:** conceptualisation, investigation, funding acquisition, writing–review and editing. **Ann‐Marie Towers:** conceptualisation, investigation, funding acquisition, writing–review and editing, formal analysis. **Freya Tracey:** investigation, writing–review and editing, formal analysis. **John Willmott:** investigation, writing–review and editing. **Claire Goodman:** conceptualisation, investigation, funding acquisition, writing–review and editing, project administration.

## Ethics Statement

The DACHA project received ethical approval for distinct elements of the research project. Work Package (WP) 2: received ethical approval from Health, Science, Engineering and Technology ECDA—University of Hertfordshire (HSK/SF/UH/04185). WP3 national care home survey: received ethical approval from Health, Science, Engineering and Technology ECDA—University of Hertfordshire (HSK/SF/UH/04301). WP5 care home pilot: received ethical approval from the London Queen's Square Research Ethics Committee (22/LO/0250). National consultation 2022: received ethical approval from Health, Science, Engineering and Technology ECDA—University of Hertfordshire (HSK/SF/UH/05009). National consultation 2023‐24: received ethical approval from Health, Science, Engineering and Technology ECDA—University of Hertfordshire (HSK/SF/UH/05487). Ethical review was not sought for this analysis of the PI process as it is not required for public involvement activity.

## Conflicts of Interest

Emily Allinson now works for the NIHR (but did not at outset of the project). This was not considered to be a conflict of interest by the DACHA team or the NIHR because her work is in an entirely separate department to the funding stream for this project. Since commencing employment at the NIHR she has not accepted remuneration from DACHA.

## Data Availability

The authors have nothing to report.

## References

[1] “DACHA Developing Resources and Minimum Data Set for Care Homes Adoption: University of Hertfordshire,” DACHA, 2024, https://dachastudy.com/.

[2] A.‐M. Towers , A. Gordon , A. T. Wolters , et al., “Piloting of a Minimum Data Set for Older People Living in Care Homes in England: Protocol for a Longitudinal, Mixed‐Methods Study,” BMJ Open 13, no. 2 (2023): e071686. 10.1136/bmjopen-2023-071686 PMC9972423 36849214

[3] J. K. Burton , A. T. Wolters , A. M. Towers , et al., “Developing a Minimum Data Set for Older Adult Care Homes in the UK: Exploring the Concept and Defining Early Core Principles,” Lancet Healthy Longevity 3, no. 3 (2022): e186–e193. 35282598 10.1016/S2666-7568(22)00010-1 PMC8901193

[4] S. Kelly , A. Cowan , G. Akdur , et al., “Outcome Measures From International Older Adult Care Home Intervention Research: A Scoping Review,” Age and Ageing 52, no. 5 (2023): afad069. 37192505 10.1093/ageing/afad069 PMC10187991

[5] G. Peryer , S. Kelly , J. Blake , et al., “Contextual Factors Influencing Complex Intervention Research Processes in Care Homes: A Systematic Review and Framework Synthesis,” Age and Ageing 51, no. 3 (2022): afac014. 35231097 10.1093/ageing/afac014 PMC8887840

[6] B. Hanratty , A. T. Wolters , A. M. Towers , et al., “Data Collection in Care Homes for Older Adults: A National Survey in England,” Journal of Long‐Term Care 2023 (2023): 288–296.

[7] “Feedback on DACHA Study's 2021 Consultation Events,” DACHA, 2021, http://dachastudy.com/wp-content/uploads/2021/10/Report-DACHA-consultation-2021.pdf.

[8] “Quality of Life Consultation Feedback Report [Internet],” DACHA, 2023, http://dachastudy.com/wp-content/uploads/2022/12/DACHA-2022-Consultation-report-FINAL-.pdf2022.

[9] “DACHA Final Consultation on Minimum Data Set—Feedback Report [Internet],” DACHA, 2024, http://dachastudy.com/wp-content/uploads/2024/05/DACHA-consultation-feedback-report-2024-v3.pdf2024.

[10] A. L. Gordon , S. Rand , E. Crellin , et al., “Piloting a Minimum Data Set for Older People Living in Care Homes in England: A Developmental Study,” medRxiv (2024), 10.1101/2024.06.07.24308589. PMC11733825 39812411

[11] A. L. Gordon , M. Franklin , L. Bradshaw , P. Logan , R. Elliott , and J. R. F. Gladman , “Health Status of UK Care Home Residents: A Cohort Study,” Age and Ageing 43, no. 1 (2013): 97–103. 23864424 10.1093/ageing/aft077 PMC3861334

[12] “A Plan for Digital Health and Social Care,” Department of Health and Social Care, Gov.UK, 2022, https://www.gov.uk/government/publications/a-plan-for-digital-health-and-social-care/a-plan-for-digital-health-and-social-care.

[13] A. Shachak , F. Buchanan , and C. Kuziemsky , “When Rules Turn Into Tools: An Activity Theory‐Based Perspective on Implementation Processes and Unintended Consequences,” Healthcare Management Forum 37, no. 3 (2024): 177–182. 38377181 10.1177/08404704241233169 PMC11044511

[14] J. Ostaszkiewicz , B. O'Connell , and T. Dunning , “Fear and Overprotection in Australian Residential Aged‐Care Facilities: The Inadvertent Impact of Regulation on Quality Continence Care,” Australasian Journal on Ageing 35, no. 2 (2016): 119–126. 26365035 10.1111/ajag.12218

[15] T. Burgher , V. Shepherd , and C. Nollett , “Effective Approaches to Public Involvement in Care Home Research: A Systematic Review and Narrative Synthesis,” Research Involvement and Engagement 9, no. 1 (2023): 38. 37268986 10.1186/s40900-023-00453-2 PMC10234794

[16] P. A. Logan , J. C. Horne , J. R. F. Gladman , et al., “Multifactorial Falls Prevention Programme Compared With Usual Care in UK Care Homes for Older People: Multicentre Cluster Randomised Controlled Trial With Economic Evaluation,” BMJ 375 (2021): e066991. 34876412 10.1136/bmj-2021-066991 PMC8649897

[17] K. Froggatt , C. Goodman , H. Morbey , et al., “Public Involvement in Research Within Care Homes: Benefits and Challenges in the APPROACH Study,” Health Expectations 19, no. 6 (2016): 1336–1345. 26620796 10.1111/hex.12431 PMC5139055

[18] O. Stirrup , G. Tut , M. Krutikov , et al., “Anti‐Nucleocapsid Antibody Levels Following Initial and Repeat SARS‐CoV‐2 Infections in a Cohort of Long‐Term Care Facility Residents in England (VIVALDI),” Wellcome Open Research 9, no. 45 (2024): 45. 38818129 10.12688/wellcomeopenres.20750.1 PMC11137476

[19] R. Stocker , K. Brittain , K. Spilsbury , and B. Hanratty , “Patient and Public Involvement in Care Home Research: Reflections on the How and Why of Involving Patient and Public Involvement Partners in Qualitative Data Analysis and Interpretation,” Health Expectations 24, no. 4 (2021): 1349–1356. 33974718 10.1111/hex.13269 PMC8369083

[20] N. Edelman and D. Barron , “Evaluation of Public Involvement in Research: Time for a Major Re‐Think?,” Journal of Health Services Research & Policy 21, no. 3 (2016): 209–211. 26510440 10.1177/1355819615612510 PMC4904347

[21] D. Burns , P. Hyde , A. Killett , F. Poland , and R. Gray , “Participatory Organizational Research: Examining Voice in the Co‐Production of Knowledge,” British Journal of Management 25 (2012): 133–144.

[22] J. Russell , N. Fudge , and T. Greenhalgh , “The Impact of Public Involvement in Health Research: What Are We Measuring? Why Are We Measuring It? Should We Stop Measuring It?,” Research Involvement and Engagement 6, no. 1 (2020): 63. 33133636 10.1186/s40900-020-00239-w PMC7592364

[23] L. Frith , “Democratic Justifications for Patient Public Involvement and Engagement in Health Research: An Exploration of the Theoretical Debates and Practical Challenges,” Journal of Medicine and Philosophy: A Forum for Bioethics and Philosophy of Medicine 48, no. 4 (2023): 400–412. 10.1093/jmp/jhad024 PMC10281369 37229555

[24] M. E. Palm , D. Evans , S. Staniszewska , et al., “Public Involvement in UK Health and Care Research 1995–2020: Reflections From a Witness Seminar,” Research Involvement and Engagement 10, no. 1 (2024): 65. 38909270 10.1186/s40900-024-00598-8 PMC11193893

[25] NIHR , Guidance on Co‐Producing a Research Project (NIHR, 2024), https://www.learningforinvolvement.org.uk/content/resource/nihr-guidance-on-co-producing-a-research-project/.

[26] A. Price , L. Albarqouni , J. Kirkpatrick , et al., “Patient and Public Involvement in the Design of Clinical Trials: An Overview of Systematic Reviews,” Journal of Evaluation in Clinical Practice 24, no. 1 (2018): 240–253. 29076631 10.1111/jep.12805

[27] “UK Standards for Public Involvement,” NIHR, 2018, https://sites.google.com/nihr.ac.uk/pi-standards/home?authuser=0.

[28] M. S. McCoy , K. R. Jongsma , P. Friesen , et al., “National Standards for Public Involvement in Research: Missing the Forest for the Trees,” Journal of Medical Ethics 44, no. 12 (2018): 801–804. 30337451 10.1136/medethics-2018-105088

[29] D. Rose , S. Carr , and P. Beresford , “‘Widening Cross‐Disciplinary Research for Mental Health’: What Is Missing From the Research Councils UK Mental Health Agenda?,” Disability & Society 33, no. 3 (2018): 476–481.

[30] B. E. Wood , R. Taylor , R. Atkins , and M. Johnston , “Pedagogies for Active Citizenship: Learning Through Affective and Cognitive Domains for Deeper Democratic Engagement,” Teaching and Teacher Education 75 (2018): 259–267.

[31] M. Hughes and C. Duffy , “Public Involvement in Health and Social Sciences Research: A Concept Analysis,” Health Expectations 21, no. 6 (2018): 1183–1190. 30159960 10.1111/hex.12825 PMC6250854

[32] B. J. Strasser , J. Baudry , D. Mahr , G. Sanchez , and E. Tancoigne , “‘Citizen Science’? Rethinking Science and Public Participation,” Science & Technology Studies. 32, no. 2 (2019): 52–76.

[33] P. Beresford and C. Slasberg , The Future of Social Care (Cheltenham, England: Edward Elgar Publishing, 2023).

[34] M. Fricker , “Epistemic Justice as a Condition of Political Freedom?,” Synthese 190, no. 7 (2013): 1317–1332.

[35] A. Banerjee , P. Armstrong , T. Daly , H. Armstrong , and S. Braedley , “‘Careworkers Don't Have a Voice:’ Epistemological Violence in Residential Care for Older People,” Journal of Aging Studies 33 (2015): 28–36. 25841727 10.1016/j.jaging.2015.02.005

[36] S. Staniszewska , J. Brett , I. Simera , et al., “GRIPP2 Reporting Checklists: Tools to Improve Reporting of Patient and Public Involvement in Research,” Research Involvement and Engagement 3, no. 1 (2017): 13. 29062538 10.1186/s40900-017-0062-2 PMC5611595

[37] K. Micklewright , A. Killett , G. Akdur , et al., “Activity Provider‐Facilitated Patient and Public Involvement With Care Home Residents,” Research Involvement and Engagement 10, no. 1 (2024): 7. 38200589 10.1186/s40900-023-00537-z PMC10782785

[38] M. Kelly , E. Allison , and K. Micklewright , “Health and Social Care Research From the Frontline: Perspectives From Care Home Staff,” Nursing and Residential Care 25, no. 11 (2023): 1–3.

[39] S. Staniszewska , J. Brett , I. Simera , et al., “GRIPP2 Reporting Checklists: Tools to Improve Reporting of Patient and Public Involvement in Research,” BMJ 358 (2017): j3453. 28768629 10.1136/bmj.j3453 PMC5539518

[40] “Where Is Information Recorded When a Person in an English Care Home Falls?,” DACHA, 2022, https://dachastudy.com/wp-content/uploads/2022/08/FINAL-DACHA-pdf.pdf2022.

[41] A.‐M. Towers , S. Allan , S. Rand , et al., “Cross‐Sectional Study Assessing the Feasibility of Measuring Residents' Quality of Life in English Care Homes and Assessing the Construct Validity and Internal Consistency of Measures Completed by Staff‐Proxy,” medRxiv (2024), 10.1101/2024.05.20.24307612. PMC11781126 39880439

[42] S. Allan , S. Rand , A.‐M. Towers , et al., “Factors Associated With Care Home Resident Quality of Life: Demonstrating the Value of a Pilot Minimum Data Set Using Cross‐Sectional Analysis From the Dacha Study,” medRxiv (2024), 10.1101/2024.05.30.24308190.

[43] A. Price , S. Schroter , R. Snow , et al., “Frequency of Reporting on Patient and Public Involvement (PPI) in Research Studies Published in a General Medical Journal: A Descriptive Study,” BMJ Open 8, no. 3 (2018): e020452. 10.1136/bmjopen-2017-020452 PMC5875637 29572398

[44] N. Curry , *Building a Resilient Social Care System in England. What Can Be Learnt From the First Wave of Covid‐19?*, Report NIHR202333 (London, England: NuffiledTrust, 2023).

[45] L. Isham , C. Bradbury‐Jones , and A. Hewison , “Reflections on Engaging With an Advisory Network in the Context of a ‘Sensitive’ Research Study,” International Journal of Social Research Methodology 22, no. 1 (2019): 67–79.

[46] B. A. Evans , A. Carson‐Stevens , A. Cooper , et al., “Implementing Public Involvement Throughout the Research Process‐Experience and Learning From the GPs in EDs Study,” Health Expectations 25, no. 5 (2022): 2471–2484. 35894169 10.1111/hex.13566 PMC9615054

[47] L. Forbat , A. Macgregor , T. Brown , et al., “Negotiating Pace, Focus and Identities: Patient/Public Involvement/Engagement in a Palliative Care Study,” Sociology of Health & Illness 46 (2024): 1327–1344. 38720523 10.1111/1467-9566.13785

[48] G. Green and T. Johns , “Exploring the Relationship (and Power Dynamic) Between Researchers and Public Partners Working Together in Applied Health Research Teams,” Frontiers in Sociology 4 (2019): 20. 33869346 10.3389/fsoc.2019.00020 PMC8022793

[49] F. Poland , G. Charlesworth , P. Leung , and L. Birt , “Embedding Patient and Public Involvement: Managing Tacit and Explicit Expectations,” Health Expectations 22, no. 6 (2019): 1231–1239. 31538704 10.1111/hex.12952 PMC6882252

[50] S. E. Knowles , D. Allen , A. Donnelly , et al., “More Than a Method: Trusting Relationships, Productive Tensions, and Two‐Way Learning as Mechanisms of Authentic Co‐Production,” Research Involvement and Engagement 7, no. 1 (2021): 34. 34059159 10.1186/s40900-021-00262-5 PMC8165763

[51] K. Plamondon , D. Banner , M. A. Cary , et al., “Relational Practices for Meaningful Inclusion in Health Research: Results of a Deliberative Dialogue Study,” Health Expectations 27, no. 1 (2024): e13865. 37749963 10.1111/hex.13865 PMC10726058

[52] T. Backhouse , A. Kenkmann , K. Lane , B. Penhale , F. Poland , and A. Killett , “Older Care‐Home Residents as Collaborators or Advisors in Research: A Systematic Review,” Age and Ageing 45 (2016): 337–345. 26790454 10.1093/ageing/afv201 PMC4846791

[53] J. Reynolds , M. Ogden , and R. Beresford , “Conceptualising and Constructing ‘Diversity’ Through Experiences of Public and Patient Involvement in Health Research,” Research Involvement and Engagement 7 (2021): 53. 34294162 10.1186/s40900-021-00296-9 PMC8295976

[54] G. Green , “Power to the People: To What Extent Has Public Involvement in Applied Health Research Achieved This?,” Research Involvement and Engagement 2, no. 1 (2016): 28. 29507763 10.1186/s40900-016-0042-y PMC5831888

[55] “Social Care Research Capacity Building Programme,” NIHR, 2024, https://arc-sl.nihr.ac.uk/events-training/what-we-offer/social-care-research-capacity-building-programme.

[56] “Public Involvement,” NHS‐HRA, 2024, https://www.hra.nhs.uk/planning-and-improving-research/best-practice/public-involvement/.

